# Proteomic signature of muscle fibre hyperplasia in response to faba bean intake in grass carp

**DOI:** 10.1038/srep45950

**Published:** 2017-04-03

**Authors:** Er-Meng Yu, Hao-Fang Zhang, Zhi-Fei Li, Guang-Jun Wang, Hong-Kai Wu, Jun Xie, De-Guang Yu, Yun Xia, Kai Zhang, Wang-Bo Gong

**Affiliations:** 1 Key Laboratory of Tropical & Subtropical Fishery Resource Application & Cultivation, Pearl River Fisheries Research Institute of CAFS, Xingyu Road No. 1, Guangzhou 510380, China; 2 State Key Laboratory of Biocontrol, School of Life Sciences, Sun Yat-sen University, Xingangxi Road, Guangzhou 510275, China

## Abstract

Fish muscle growth is important for the rapidly developing global aquaculture industry, particularly with respect to production and quality. Changes in muscle fibre size are accomplished by altering the balance between protein synthesis and proteolysis. However, our understanding regarding the effects of different protein sources on fish muscle proteins is still limited. Here we report on the proteomic profile of muscle fibre hyperplasia in grass carp fed only with whole faba bean. From the results, a total of 99 significantly changed proteins after muscle hyperplasia increase were identified (*p* < 0.05, ratio <0.5 or >2). Protein–protein interaction analysis demonstrated the presence of a network containing 56 differentially expressed proteins, and muscle fibre hyperplasia was closely related to a protein–protein network of 12 muscle component proteins. Muscle fibre hyperplasia was also accompanied by decreased abundance in the fatty acid degradation and calcium signalling pathways. In addition, metabolism via the pentose phosphate pathway decreased in grass carp after ingestion of faba bean, leading to haemolysis. These findings could provide a reference for the prevention and treatment of human glucose-6-phosphate dehydrogenase deficiency (“favism”).

Grass carp (*Ctenopharyngodon idella*) is currently the largest globally produced aquaculture species, with an annual production of more than 5 million tonnes in China alone. It is not only an important source of low-cost protein in developing or undeveloped regions, but it also makes a significant contribution to the supply of high-quality animal protein in the world^1^. From the perspective of global marine fishery resources, grass carp has a high potential for sustainable development worldwide due to its low requirement on fish meal^2^. In China, grass carp farming has long provided an effective livelihood for low-income farmers and aquaculturists. With social and economic development, however, grass carp farmers are no longer satisfied with the low market value for the bulk products, and are thus attempting to promote the grass carp as a high-value commodity product.

The introduction of crisp grass carp variety (*Ctenopharyngodon idellus* C.et V) was one of the most important developments in the carp aquaculture industry. In the early 1970s, fish farmers in Dongsheng Town, Guangdong Province, China, accidentally discovered that the muscle structure of grass carp changed significantly after they were fed with faba bean (*Vicia faba* L.) solely for 90–120 days. Unlike ordinary grass carp, this carp variety when fed with faba bean exhibited a significant increase in muscle hardness and crispiness, and thus became popular among local people^3^.

The change in grass carp muscle after feeding with faba bean is not an accidental phenomenon, and an increase in muscle hardness has also been found in channel catfish *(Ictalurus punctatus*)^4^, crucian carp (*Carassius auratus*)^5^ and tilapia (*Oreochromis mossambicus*)^6^. Faba bean not only increases muscle hardness in fish, but also in birds and mammals^7^. Despite the complex nutrients in faba bean, it has the same effect on muscle regulation in many different animals, indicating that the signalling pathway for the nutrition regulation of faba bean exhibits a relatively convergent molecular mechanism. In view of this, we previously compared the gene expression profiles of crisp grass carp and ordinary grass carp, and found 241 differentially expressed genes in the muscle of crisp grass carp, including six collagen genes, four myocyte differentiation genes and fourteen cytoskeletal organisation genes^8^. However, only 20–40% of the protein concentrations are determined by the corresponding mRNA concentrations^9^. Among the higher vertebrates, muscle proteomic information is available for humans^10^ and rats^11^, and for lower vertebrates such as zebrafish^12^ and Catla fish^13^. A few studies have focused on nutrition-related proteomic mechanisms in teleosts; however, only the differences in muscle proteins after the addition of amino acid (lysine)^14^ and nucleotide^15^ have been analysed. Differences in muscle proteomics between wild and farmed fish, and the nutrition-related cytoplasm proteomic mechanisms in 15 species of wild marine fish, have also been analysed^16^.

The present study focused on distinctive nutritional regulation, in which grass carp were fed faba bean without other nutritional supplements, and the relevant regulation mechanisms were then investigated by proteomics.

In fish, the biological pathways regulating the processes driving a population of myogenic cells to follow a hyperplastic or hypertrophic programme are still poorly understood. Furthermore, the metabolic mechanisms described in mammals are not always valid for teleosts^17^. Our previous microstructure observations found an increased number of muscle fibres in the muscle of crisp grass carp compared with that of ordinary grass carp^18^. Despite a significantly decreased fibre diameter, crisp grass carp still gained weight at a low growth rate, without obvious muscle atrophy. Hence, we speculated that the muscle of crisp grass carp exhibits muscle fibre hyperplasia. Therefore, exploring the mechanism of muscle fibre hyperplasia in crisp grass carp will provide important guidance in fish muscle fibre research.

The main aim of the present study was to use faba bean to investigate the molecular signalling pathways regulating muscle fibre hyperplasia in grass carp. Currently, stable isotope labelling and high-performance liquid chromatographic–tandem mass spectrometry (iTRAQ-LC-MS/MS) is an effective method for investigating intracellular protein composition and content changes due to its complete labelling, high sensitivity and good reproducibility^19^. We investigated proteins both qualitatively and semi-quantitatively, and the metabolic pathways of protein expression levels and protein–protein interaction networks were also analysed. Furthermore, the regulatory mechanisms of muscle fibre hyperplasia in grass carp fed with faba bean were explored by comparing the proteomic data with previous gene expression profiling data. The results of this study will provide a theoretical basis for the nutritional regulation of muscle in other fish species. In addition, given the current lack of scientific data for the application of nutritional supplements in improving human bodybuilding and increasing muscle strength, this study might provide a potential reference for the nutritional regulation of human muscle (including in bodybuilding, exercise and disease).

## Results

### Muscle hardness and chewiness, and muscle fibre diameter and density

**Figure 1:**
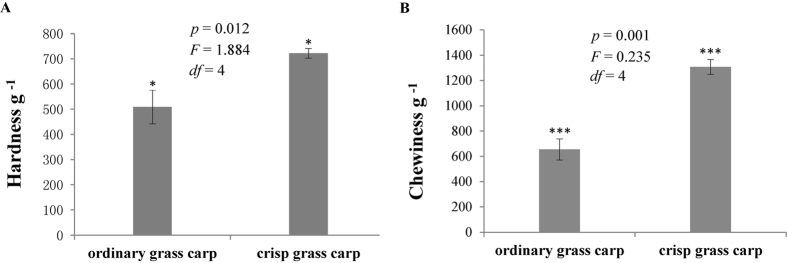
Muscle hardness (**A**) and chewiness (**B**) of crisp grass carp and ordinary grass carp. Independent-samples Tukey’s text was carried out for the two independent samples, and *p* and *F* values were accurately calculated (95% confidence levels). Datasets are presented as mean values, and error bar represents its standard deviation. *p < 0.05, **p < 0.01, ***p < 0.001.

The muscle hardness and chewiness results for crisp and ordinary grass carp are shown in Fig. 1. The hardness of ordinary grass carp was 508.52 ± 66.89 g, and that of crisp grass carp was 721.97 ± 19.14 g. The chewiness of ordinary grass carp was 655.14 ± 83.66 g, and that of crisp grass carp was 1307.44 ± 58.86 g. Compared with the ordinary grass carp, the hardness and chewiness of crisp grass carp exhibited a 41.97% and 99.57% increase (*p* < 0.05).

**Figure 2:**
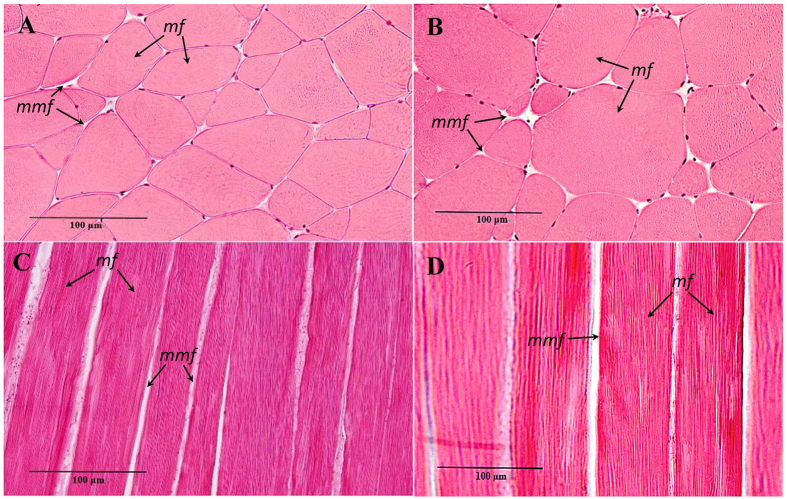
Microstructure observation of grass carp muscle (×400). Transverse section microstructure of crisp grass carp (**A**). Transverse section microstructure of ordinary grass carp (**B**). Longitudinal section microstructure of crisp grass carp (**C**). Longitudinal section microstructure of ordinary grass carp (**D**). Haematoxylin and eosin stainings was used, and microstructure observations were by light microscopy, mf, muscle fibre; mmf, matrix between muscle fibres.

As shown in the transverse muscle microstructure diagrams obtained at the same magnification and field of view (Fig. 2), crisp grass carp exhibited increased muscle fibre numbers and decreased muscle fibre diameters compared with those of the control group. Statistical analysis showed that the muscle fibre densities of crisp grass carp and ordinary grass carp were 231 ± 14 and 116 ± 11 fibres mm^−2^ respectively, and the muscle fibre diameters of crisp grass carp and ordinary grass carp were 75.17 ± 1.92 and 104.58 ± 6.54 μm respectively. Compared with ordinary grass carp, the muscle fibre density of crisp grass carp increased significantly and the diameter decreased significantly (*p* < 0.05).

### Haematological parameters

**Figure 3:**
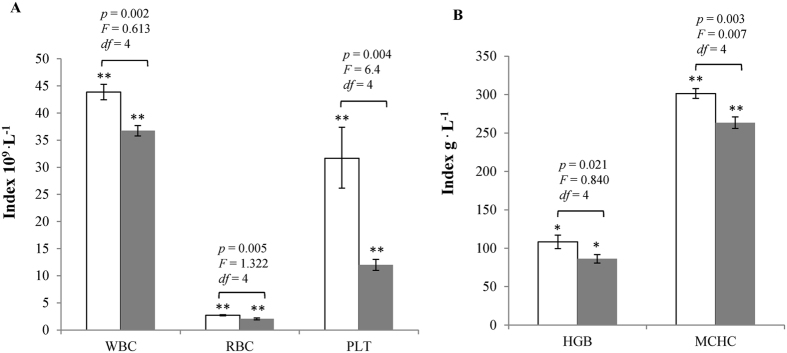
Hematological parameters of crisp grass carp and ordinary grass carp. (**A**) White blood cell counts (WBC), red blood cell counts (RBC) and platelet counts (PLT). (**B**) Haemoglobin (HGB) and mean corpuscular haemoglobin (MCHC) concentrations. “□” Denote ordinary grass carp. and “
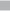
” denote crisp grass carp. Independent-samples Tukey’s text was carried out for the two independent samples, and *p* and *F* values were accurately calculated (95% confidence levels). Datasets are presented as mean values, and error bar represents its standard deviation. *p < 0.05, **p < 0.01, ***p < 0.001.

The blood indices for crisp and ordinary grass carp are shown in Fig. 3. The white blood cell counts of ordinary and crisp grass carp were 43.87 ± 1.43 cells × 10^9^ L^−1^ and 36.73 ± 0.96 cells × 10^9^ L^−1^; the red blood cell counts of ordinary and crisp grass carp were 2.71 ± 0.096 cells × 10^9^ L^−1^ and 2.05 ± 0.18 cells × 10^9^ L^−1^; and the platelet counts of ordinary and crisp grass carp were 31.67 ± 5.69 cells × 10^9^ L^−1^ and 12.00 ± 1.00 cells × 10^9^ L^−1^. The haemoglobin concentration of ordinary grass carp was 108.33 ± 8.74 g L^−1^, and that of crisp grass carp was 86.33 ± 5.51 g L^−1^. The mean corpuscular haemoglobin concentrations of ordinary and crisp grass carp were 301.33 ± 6.35 and 263.33 ± 7.51 g L^−1^. All of the blood parameters and index for crisp grass carp were significantly lower than those for ordinary grass carp (*p* < 0.05).

### Differentially expressed protein analysis

Comparing the experimental and control groups, 99 proteins showed at least twofold differential expression with *p* < 0.05, and 84 proteins had Gene Ontology (GO) annotations. Table 1 shows the proteins related to the cytoskeleton, muscle fibre development, collagen, and calcium ion binding. A total of 21 significantly differentially expressed proteins were found, with six upregulated and 15 downregulated in the muscle of crisp grass carp (*p* < 0.05).

Table 2 shows the 71 differentially expressed proteins associated with metabolic processes, including redox activity, biosynthesis, lipid metabolism and other physiological processes. Compared with ordinary grass carp, 16 structural proteins in the muscle of crisp grass carp showed significantly higher expression and 55 showed significantly lower expression (*p* < 0.05). In addition, three proteins (ENSDARG00000043403, zgc:56585, and ENSDARG00000009567) of unknown function were found.

The expression of 41 proteins, including membrane, nucleus, mitochondria and other organelle proteins, are summarised in Table 3. Compared with ordinary grass carp, 12 proteins exhibited greater than two times change, and 29 had less than 0.5 times change in the muscle of crisp grass carp (*p* < 0.05). In addition, three proteins (loc100691952, loc101068541 and loc794259) of unknown function were found.

### Protein–protein interaction network

**Figure 4:**
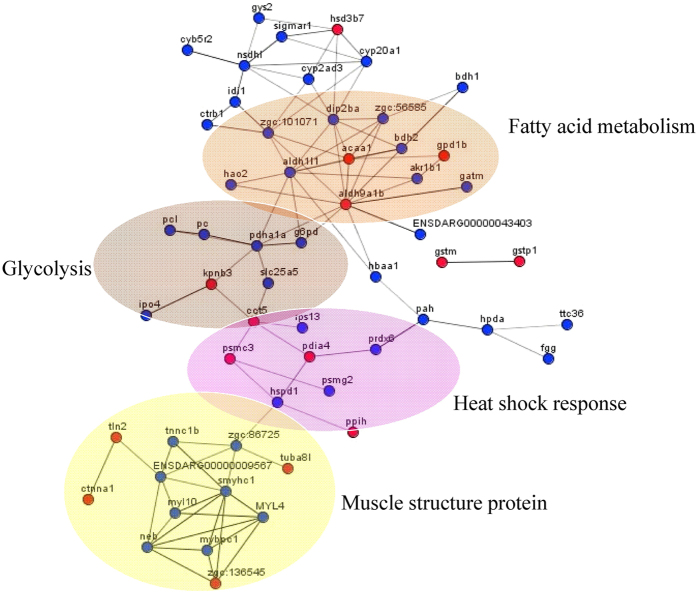
Protein-protein interaction network of muscle fibre development and function. “
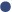
” Denote down-regulated proteins, and “
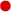
” denote upregulated proteins. The Lines “—” indicate that there was an interaction between proteins. Proteins: gys2, glycogen synthase 2; hsd3b7, hydroxy-delta-5-steroid dehydrogenase, 3; sigmar1, sigma non-opioid intracellular receptor 1; cyb5r2, NADH-cytochrome b5 reductase 2; nsdhl, NAD(P)-dependent steroid; cyp20a1, cytochrome P450, family 20, subfamily A, polypeptide 1; cyp2ad3, cytochrome P450 2AD3; idi1, isopentenyl-diphosphate Delta-isomerase 1; ctrb1, chymotrypsin B1 precursor; zgc:101071, acyl-CoA synthetase long-chain family member 1; dip2ba, DIP2 disco-interacting protein 2 homolog B; bdh1, 3-hydroxybutyrate dehydrogenase, type 1; bdb2, RecName: Full = 3-hydroxybutyrate dehydrogenase type 2; acaa1, acetyl-coenzyme A acyltransferase 1; aldh1l1, aldehyde dehydrogenase 1 family, member L1; hao2, hydroxyacid oxidase 2; aldh9a1b, aldehyde dehydrogenase 9 family, member A1b; akr1b1, aldo-ketoreductase family 1, member B1; gpd1b, glycerol-3-phosphate dehydrogenase 1b; gatm, RecName: Full = glycine amidinotransferase, mitochondrial; g6pd, glucose-6-phosphate 1-dehydrogenase; pdha1a. pyruvate dehydrogenase (lipoamide) alpha 1a; pc, pyruvate carboxylase; pcl, pyruvate carboxylase, like; kpnb3, karyopherin (importin) beta 3; slc25a5, solute carrier family 25 alpha, member 5; cct5, chaperonin-containing TCP1, subunit 5; ipo4, PREDICTED: importin-4; ips13, 40 S ribosomal protein S13; psmc3, proteasome (prosome, macropain) 26 S subunit, ATPase, 3; pdia4, protein disulfideisomerase associated 4; prdx6, peroxiredoxin 6; psmg2, Chaperone protein promoting assembly of the 20 S proteasome as part of a heterodimer with psmg1; hspd1, 60 kDa heat shock protein 1; ppih, peptidyl-prolyl cis-trans isomerase H; gstm, glutathione *S*-transferase M; gstp1, glutathione *S*-transferase pi 1; hbaa1, haemoglobin alpha adult-1; pah, phenylalanine hydroxylase; hpda, 4-hydroxyphenylpyruvate dioxygenase a; ttc36, tetratricopeptide repeat domain 36; fgg, fibrinogen gamma polypeptide; tln2, PREDICTED: talin-2-like; ctnna1, PREDICTED: catenin alpha-1-like; tnnc1b, troponin C type 1b (slow); zgc:86725, muscle actin type 1; myl10, myosin, light chain 10, regulatory; smyhc1, myosin heavy chain embryonic type 3; neb, novel protein similar to vertebrate nebulin (NEB); mybpc1, myosin-binding protein C, slow-type; myl4, myosin, light chain 4; tuba8l, tubulin, alpha 8 like; zgc:136545, myosin-binding protein C, fast type a; zgc:56585, ENSDARG00000043403, ENSDARG00000009567 are unknown proteins.

A protein**–**protein interaction network diagram consisting of the 56 differentially expressed proteins, associated with muscle development and function, was generated (Fig. 4). The network was divided into four parts, based on the structure and function of the proteins: the lipid metabolism protein network, the glucose metabolism protein network, the heat shock response protein network and the muscle fibre composition protein network. ACAA1 (thiolase) is a key enzyme in the fatty acid metabolism pathway. From the protein**–**protein interaction network, this enzyme was shown to interact with gc:101071 (long-chain fatty acid synthase), BDH2 (3-hydroxybutyrate dehydrogenase), ALDH9A1B (aldehyde dehydrogenase), GPD1B (3-glycerophosphate dehydrogenase), HAO2 (hydroxyacid oxidase) and other key enzymes in lipid metabolism. These proteins cooperated to form a lipid metabolism protein**–**protein interaction network centred on ALDH9A1B and ACAA1. In the glucose metabolism protein network, G6PD (glucose-6-phosphate dehydrogenase), PDHA1A (pyruvate dehydrogenase), PC1 (pyruvate carboxylase), SLC25A5 (soluble carrier family), KPNB3 (cytoplasmic importin) and other proteins formed a protein**–**protein interaction network centred on PDHA1A (pyruvate dehydrogenase). Lipid metabolism and glucose metabolism were connected by interaction between the centre of the lipid metabolism network, ALDH9A1B, and the centre of the glucose metabolism network, G6PD. The results also showed that RPS13 (40 S ribosomal protein), PSMC3 (26 S proteasome regulatory subunit), HSPD1 (heat shock protein), and other proteins formed a heat shock response protein**–**protein interaction network. This network connected the glucose metabolism network and the muscle fibre composition protein network (Fig. 4) via KPNB3 and HSPD1. The muscle fibre composition protein network was mainly composed of TNNC1B (troponin C type 1b), SMYHC1 (myosin heavy chain embryonic type 3), MYL10 (myosin light chain 10, regulatory), MYBPC1 (myosin-binding protein C, slow type), NEB (novel protein similar to vertebrate nebulin), MYL4 (myosin, light chain 4), zgc:86725 (muscle actin type 1), zgc:136545 (myosin-binding protein C, fast type a), and ENSDARG00000009567. As shown in Fig. 4, lipid and glucose metabolism are closely related, and can be connected with the muscle fibre composition protein network via heat shock response, thus forming a protein**–**protein interaction network of muscle metabolism and structure.

**Figure 5:**
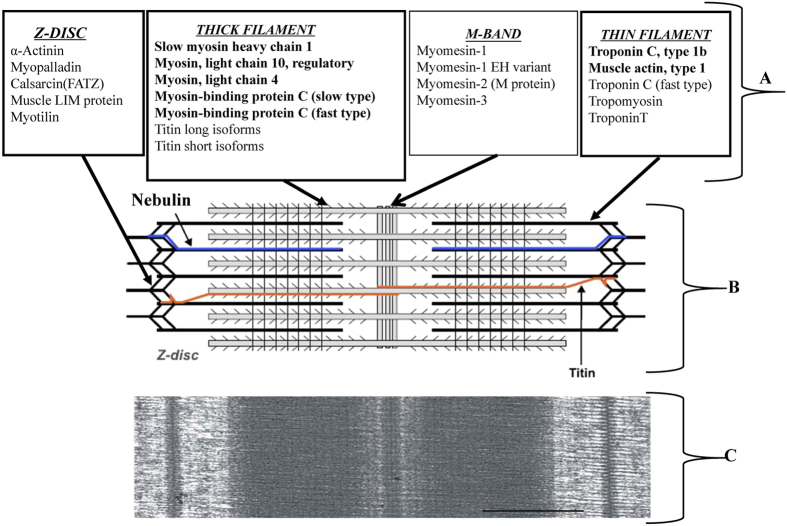
Schematic view showing the main components of the striated muscle sarcomere. (**A** and **B**) and transmission electron micrograph showing the appearance of the sarcomere in longitudinal section (**C**). The molecular components known to exist as multiple isoforms with differential distribution are indicated (A and B modified from Schiaffino and Reggiani (2011)^69^; C is from Luther (2009)^70^). The expression of these proteins (including the following: slow myosin heavy chain 1 (smyhc1); myosin, light chain 10, regulatory (myl10); myosin, light chain 4 (myl4); myosin-binding protein C (slow type) (mybpc1); troponin C, type 1b (tnnc1b); muscle actin, type 1 (zgc:86725); nebulin (neb)) was downregulated. The expression of myosin-binding protein C (fast type) (zgc:136545) was upregulated.

The differentially expressed proteins of the main components of the striated muscle sarcomere are shown in Fig. 5, and included SMYHC1, MYL10, MYL4, MYBPC1 (slow type), MYBPC1 (fast type), TNNC1B, muscle actin type 1 and Nebulin.

### Signal transduction pathway

**Figure 6:**
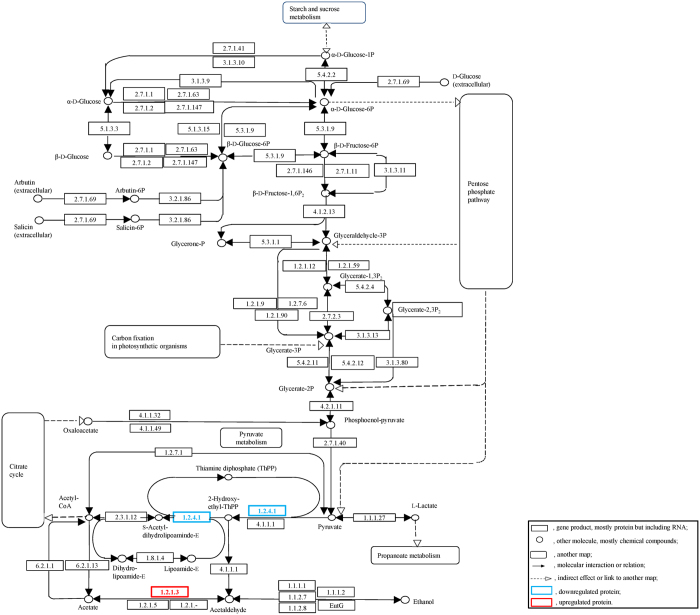
Pathway of glycolysis/gluconeogenesis. Box 1.2.4.1 is the pyruvate dehydrogenase E1 component, and box 1.2.1.3 is the aldehyde dehydrogenase (NAD^+^). This diagram was formed from differentially expressed proteins that were analysed using WebLinks of the KEGG system^66^ and KEGG database (http://www.kegg.jp/dbget-bin/www_bget?map00010).

**Figure 7:**
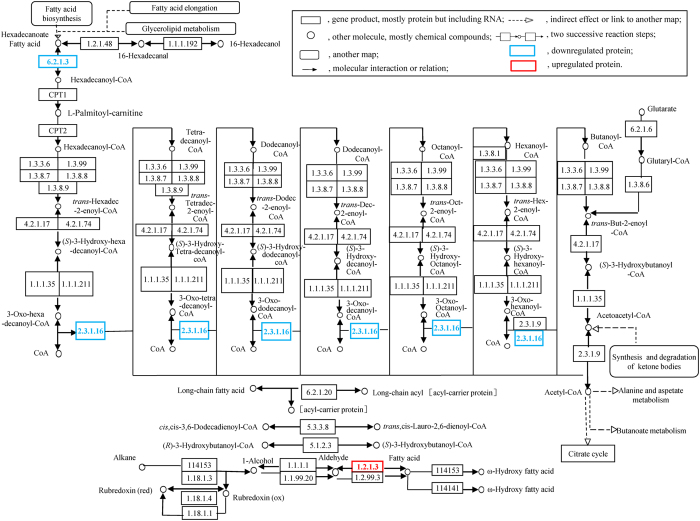
Pathway of fatty acid degradation. Box 6.2.1.3 is long-chain acyl-CoA synthetase, Box 2.3.1.16 is acetyl-coenzyme A acyltransferase and box 1.2.1.3 is aldehyde dehydrogenase (NAD^+^). This diagram was formed from differentially expressed proteins that were analysed using WebLinks of the KEGG system^66^ and KEGG database (http://www.kegg.jp/dbget-bin/www_bget?map00071).

Figure 6 shows the differentially expressed proteins involved in the glycolytic metabolic pathway, including proteins involved in the whole process of glycolysis and the metabolic cycle. Significant differences were found in the protein expressions of crisp grass carp and ordinary grass carp for the pyruvate dehydrogenase E1 component and aldehyde dehydrogenase (NAD^+^) (*p* < 0.05). Figure 7 depicts the fatty acid degradation pathway. The protein expression of aldehyde dehydrogenase (NAD^+^) was 88-fold higher for crisp grass carp compared with ordinary grass carp.

## Discussion

The “crisp” quality of the flesh of crisp grass carp after cooking is a typical characteristic of this fish variety^3^. The significantly increased chewiness of crisp grass carp muscle compared with that of ordinary grass carp is an important contributor to this “crispness”. Compared with ordinary grass carp, crisp grass carp has significantly increased muscle hardness and muscle fibre density. Wild sea bass (*Dicentrarchus labrax*)^20^ and Atlantic salmon (*Salmo salar*)^21^ also show a significant increase in muscle hardness and density over that of less-active farmed fish, and the number and size distribution of muscle fibres are significantly correlated with texture attributes. Fish quality is wide in its definition, and depends on the species in question and the relevant market. Crisp grass carp has a similar flavour to that of wild active fish, so its “crisp” texture is an important and valuable attribute.

In fish species, muscle growth is the result of hyperplasia (an increase in myofibre number throughout life) and hypertrophy (an increase in myofibre size)^22^. This is in contrast to muscle growth in land animals in which muscle growth is predominantly due to hypertrophy. Individual fish species exhibit unique combinations of hypertrophy and hyperplasia during postnatal muscle growth. These fish exhibit the same differences in muscle hyperplasia versus hypertrophy during muscle regeneration^23^. There is growing evidence that, at least in grass carp, sufficient faba bean will stimulate hyperplasia, but not hypertrophy. In the present study, muscle fibre density significantly increased in grass carp fed with whole faba bean solely, compared with formulated feed, and the fish weight was gained at a low growth rate, suggesting that the muscle growth of crisp grass carp is the result of hyperplasia.

In fish, the biological pathways regulating myogenic cell populations by a hypertrophic or a hyperplastic programme are still seldom understood. In this study, a total of 99 differentially expressed proteins (ratio <0.5 or >2) were detected in the muscle of crisp grass carp and ordinary grass carp, and the greatest increase in expression was 88-fold. In our previous transcriptome results, the largest difference in mRNA expression was 11-fold, indicating that the proteome had more significant differential expression than that of the transcriptome^8^. In this study, a protein–protein interaction network of 56 differentially expressed proteins was generated. This network included the lipid metabolism protein network, the glucose metabolism protein network, the heat shock response protein network and the muscle fibre composition protein network. Lipid metabolism and glucose metabolism were closely linked via interaction between aldh9a1b and g6pd, and lipid metabolism and glucose metabolism were connected with the muscle fibre composition protein network via the heat shock response, thus forming a protein–protein interaction network of muscle metabolism and structure.

The maintenance and completion of muscle fibre growth and development often require the participation of a variety of proteins in the form of an interacting protein network. However, only a few studies have investigated protein–protein interaction in fish muscle development, with the most research focussing on the changing trends of individual proteins. Salem *et al*. examined proteome expression changes in the muscle of rainbow trout (*Oncorhynchus mykiss*), but did not establish a protein–protein interaction network associated with muscle development^9^. In investigating sarcoplasmic fish proteomes, Carrera *et al*. established a muscle development protein network composed of seven proteins via protein–protein interaction analysis^16^. In the present study, a muscle development protein–protein interaction network consisting of 12 proteins, including structural proteins (talin 2, catenin (a1), and tubulin (a8)), thick filament composition proteins (myosin heavy chain, myosin light chain 4, myosin light chain 10, myosin-binding protein C (slow type) and myosin-binding protein C (fast type), thin filament composition proteins (actin, troponin and nebulin) and an unknown protein were found.

Talin plays an important role in muscle cell proliferation and the formation of multinucleated muscle fibres (myotubes) by promoting the formation of new muscle fibres^24^. We found the expression of talin in the muscle of crisp grass carp to be significantly increased, suggesting that it plays an important role in muscle fibre hyperplasia in crisp grass carp. In early zebrafish embryos, overexpression of the Wnt/β-catenin signalling pathway has been shown to cause muscle hyperplasia^25^. In the current study, the expressions of catenin and talin, which are key signalling factors in the Wnt/β-catenin signalling pathway, was significantly increased in crisp grass carp. This change might result in an increased number of muscle fibres, and thus induce muscle fibre hyperplasia through the synergy of related signalling factors.

Myosin, an important structural protein that forms thick filaments, is composed of myosin heavy chain (MHC) and myosin light chain (MLC). MHC comprises two subunits^26^. MLC also contains two subunits–essential light chain (ELC) and regulated light chain (RLC)^27^–that bind closely to the two subunits of MHC. ELC binds directly to MHC, and plays an important role in maintaining the ATPase activity of MHC and is unaffected by the matrix calcium concentration^26^. Decreased protein expression of MHC and MLC will result in a decrease in myosin ATPase activity and conformational changes of MHC, which further lead to diminishing space between thick and thin filaments and a decrease in the muscle fibre diameter^28^. The current study also found decreased protein expressions of MHC and MLC in the muscle of crisp grass carp. Moreover, both this study and another confirmed that the muscle fibre diameter was smaller in crisp grass carp than in ordinary grass carp^17^. Thus, we speculated that myosin played an important role in the decrease of the muscle fibre diameter.

Myosin-binding protein C (MBPC) is present in the sarcomere A-band. It is involved in the regulation of myosin filament polymerisation^29^; plays an important role in the interaction between thick and thin filaments; and is also involved in the polymerisation and extension of actin-filament-extending protein, prompting the extension of titin from the thick filaments to the sarcomere Z-line^30^. In the current study, the decreased MBPC expression in the muscle of crisp grass carp might lead to reduced Z-line spacing. It has also been demonstrated that MBPC might have actin-binding sites^31^, and might extend from the thick filament rod to the thin filament and construct a bridge connecting the thick and thin filaments in conjunction with MHC^32^. Calcium-ion-dependent protein kinase phosphorylation sites are present in the N-terminus of MBPC. When the calcium concentration increases within the body, protein kinase phosphorylates and activates MBPC via these phosphorylation sites. The phosphorylation of MBPC can increase the width of the cross-bridge connecting the thick and thin filaments, which can result in an increased muscle fibre diameter^33^. In the current study, the calcium-signalling pathway was found to be downregulated in the muscle of crisp grass carp. We speculated that calcium-ion-dependent MBPC protein kinase activity was decreased, resulting in a decrease in the phosphorylation of MBPC. This change might lead to the decrease in the muscle fibre diameter in crisp grass carp compared with that of ordinary grass carp.

Monomers of actin polymerise into 5–8-nm-diameter helical actin filaments, and constitute a network of muscle fibres in conjunction with myosin filaments. The muscle fibre diameter and density are both directly affected by the actin content^34^. The current study found that actin expression in the muscle of crisp grass carp was significantly reduced, suggesting that the diameter of muscle fibres constructed of actin monomers might be decreased. The expression of nebulin, an important protein-regulating actin, was also significantly decreased in crisp grass carp. Nebulin is composed of an N-terminal repeat domain, a linker domain and a C-terminal Src homology-3 (SH3) domain. The central portion of the SH3 domain is a hydrophobic crack constructed by the orthogonal packing of two beta-pleated sheets. Sheet can bind to the SH3-binding sites of titin or myopalladin (polyproline type II helices), and plays an important role in the formation of the Z-line cross-bridge in the sarcomere (a structural unit of myofibril)^35^. Furthermore, many SH3-binding sites were present in the PEVK-independent domain, and a large number of nebulin molecules could be recruited to the complexes after the SH3-binding sites were bound to the C-terminal SH3 domain of nebulin, thus promoting nebulin to extend continuously to the Z-line, and then become tightly anchored to the myopalladin of the Z-line^36^. In this process, nebulin, together with titin, myopalladin and other proteins, is involved in the formation of the sarcomere Z-line. The decreased expression of nebulin will lead to a decrease in both the Z-line width and the muscle fibre diameter^37^.

Compared with ordinary grass carp, talin 2, catenin (a1), tubulin (a8) and myosin-binding protein C (fast type) were upregulated in the muscle of crisp grass carp, whereas myosin heavy chain, myosin light chain 4, myosin light chain 10, troponin, actin, nebulin, myosin-binding protein C (slow type), and the unknown protein ENSDARG00000009567 were significantly downregulated. The interaction between the 12 proteins (protein–protein interaction network) demonstrated how faba bean decreased the muscle fibre diameter and increased the muscle fibre density of crisp grass carp.

The current study showed that the expression of glucose-6-phosphate dehydrogenase (G6PD) in the muscle of crisp grass carp decreased to 3% that of ordinary grass carp. Earlier research also demonstrated that levels of G6PD are significantly lower in the muscle of crisp grass carp than in that of ordinary grass carp^38^. G6PD deficiency is a human genetic disease with high incidence^39^ caused by *G6pd* gene deletion. G6PD is the enzyme that catalyses the first reaction step of the pentose phosphate pathway. NADPH produced from this reaction, in conjunction with glutathione reductase, transforms oxidised glutathione into reduced glutathione, which plays an important role in maintaining the metabolism and membrane integrity of red blood cells. As red blood cells do not contain mitochondria, the pentose phosphate pathway involving G6PD is the only way to maintain their metabolism and integrity. G6PD deficiency can lead to serious damage to red blood cell membranes^40^. It has been shown that faba bean intake can cause haemolysis (rupture of red blood cells and release of haemoglobin) in humans with G6PD deficiency. This is mainly because faba bean contains levodopa, which is converted to dopaquinone under the influence of the threonine enzyme. Dopaquinone can produce oxidised glutathione by binding to reduced glutathione, thereby further reducing levels of glutathione – a phenomenon known as favism^40^. The current study also found that NADH-cytochrome b5 reductase regulating NADP was significantly downregulated, and the expression of glutathione reductase interacting with NADPH was also decreased. Thus, we speculated that the decrease in G6PD and NADH-cytochrome b5 reductase led to the reduction of NADPH in the muscle of crisp grass carp. Moreover, as the expression of glutathione reductase interacting with NADPH was decreased, we further speculated that the production of reduced glutathione decreased, thereby affecting the metabolism and membrane integrity of red blood cells.

The expression of glyceraldehyde 3-phosphate dehydrogenase in the muscle of crisp grass carp was significantly increased, by 10-fold. The increased expression of this enzyme can lead to reduced accumulation of dihydroxyacetone phosphate^41^. Dihydroxyacetone phosphate is an important intermediate product of the pentose phosphate pathway. Plasmalogen produced from the substrate reaction is mainly stored in red blood cells, and its level reflects the number of red blood cells to a certain extent^42^. Moreover, plasmalogen and platelet-activating factor (PAF) have a very similar structure. PAF can increase vascular permeability, and may also be related to red blood cell haemolysis. It plays a regulatory role in inflammation and haemolytic complications in many diseases^43^. In the current study, the upregulated expression of glycerol-3-phosphate dehydrogenase resulted in decreased levels of dihydroxyacetone phosphate. This decrease might lead to further reduction in plasmalogen expression, resulting in decreased regulation of haemolysis in patients with favism. The current study also found significantly decreased expressions of pyruvate carboxylase and fumarylacetoacetase in the muscle of crisp grass carp. Pyruvate carboxylase can synthesise oxalacetic acid from pyruvic acid. Fumarylacetoacetase can break down 4-fumarylacetoacetic acid into fumaric acid and acetoacetic acid. Oxalacetic acid and fumaric acid are important substrates for the formation of haemoglobin. The degradation or reduction of haem can cause premature apoptosis of red blood cells^44^. Thus, the decrease in pyruvate carboxylase and fumarylacetoacetase in the muscle of crisp grass carp could lead to premature apoptosis of red blood cells.

Plant ingredients contain various anti-nutritional factors that can have adverse effects in fish^45^. The current study indicated that feeding of faba bean reduced the expression of g6pd in grass carp, and decreased the expressions of NADH-cytochrome b5 reductase, glutathione reductase, pyruvate carboxylase, and fumarylacetoacetase, and increased the expression of glycerol-3-phosphate dehydrogenase. The changes in these enzymes might lead to inhibited metabolism, damaged membranes, and premature apoptosis of red blood cells, thus resulting in haemolysis. Through long term feeding of faba bean, the crisp grass carp feeding excessive faba bean were demonstrated to experience haemolysis and even death, which have also been reported by Tan and Li^46^. Data from the current study could be of use in the prevention and treatment of human G6PD deficiency (favism).

The glycolytic pathway is a common metabolic pathway that derives energy from glycogen for all animal, plant and microbial cells. The proteomic data obtained in the current study indicated that two enzymes involved in the intermediate process of glycolysis were significantly upregulated in the muscle of crisp grass carp, with the protein expression of the aldehyde dehydrogenase 9 family, for example, increasing 87.90-fold. In the expression profile of crisp grass carp, we also found that the gene expression of aldehyde dehydrogenase increased 5.51-fold^8^. Under anaerobic conditions, aldehyde dehydrogenase can oxidise glyceraldehyde-3-phosphate to 1,3-diphosphoglycerate, and reduce NAD^+^ to NADH. This is the only anaerobic reaction associated with ATP generation in the glycolytic pathway^47^. This indicated that crisp grass carp needed to accelerate glucose metabolism, to provide more energy under anaerobic or anoxic conditions. Thus, crisp grass carp might be less tolerant of hypoxia than ordinary grass carp. Prior research also found that the dissolved oxygen threshold in the water was 1.68 mg L^−1^ for crisp grass carp and 0.54 mg L^−1^ for ordinary grass carp^46^.

Under aerobic conditions, the intermediate product of glycolysis (pyruvate) enters the citric acid cycle via pyruvate dehydrogenase, to provide energy via oxidative decomposition. This study showed significantly decreased protein expression of pyruvate dehydrogenase, which is associated with the decomposition of pyruvate. In our previous study, the gene expression of pyruvate dehydrogenase also decreased 0.15-fold in the crisp grass carp^8^. This indicated that during muscle hardening of grass carp, pyruvate less efficiently entered the mitochondria, to undergo oxidative decomposition via the citric acid cycle pathway. This suggested that this glycolysis product in the muscle cells of crisp grass carp underwent oxidative decomposition predominantly through the anaerobic metabolism pathway, i.e., the anaerobic metabolism accelerated. Similar experiments have been carried out on other fish species, with the anaerobic metabolism found to accelerate with increasing muscle hardness in Atlantic salmon^48^. In addition, the glycolytic pathway has also been investigated in pork of different qualities, revealing that the expression of glyceraldehyde-3-phosphate dehydrogenase and lactate dehydrogenase is significantly increased in pork with a hard texture^49^. However, the specific mechanism of the glycolytic metabolic pathway during muscle hardening remains to be elucidated.

Energy released from glycolysis is used for the synthesis and metabolism of fat, in addition to other activities such as growth and development. Glycerol-3-phosphate dehydrogenase catalyses the glycolytic intermediate dihydroxyacetone phosphate, to form glycerol-3-phosphate, which further reacts with fatty acyl-coenzyme A (acyl-CoA) to synthesise triacylglycerols. Most fatty acids in the body are stored in the form of triacylglycerols^50^. The current study found that the expression of glycerol-3-phosphate dehydrogenase increased 10-fold, indicating that energy released from the glycolytic pathway was stored in fatty acids in the muscle of crisp grass carp to a greater extent than in ordinary grass carp. It has been reported that energy mainly comes from the lipid metabolic pathways when the body is hungry, and in this study, stored lipid probably provide more energy for life activities of crisp grass carp when it was hungry^51^.

Fat is first decomposed into fatty acids and glycerine, and these fatty acids then enter the muscle, brain and other tissues, and are degraded to provide energy for growth and development^52^. The first reaction in the degradation of fatty acids is their activation, i.e., the conversion of fatty acids to acyl-CoA. This reaction is catalysed by long-chain acyl-CoA synthetase, and consumes ATP^53^. The decreased expression of long-chain acyl-CoA synthetase in this study suggested that fatty acid degradation is slow in the muscle of crisp grass carp. Acyl-CoA formed by the degradation of fatty acids is transported via the acyl-carnitine or carnitine transporter to the mitochondria for oxidation. Fatty acid oxidation usually occurs via β-oxidation^54^, and acetyl-CoA is formed by β-oxidation after dehydrogenation, hydration, re-dehydrogenation and thiolysis^55^. Thiolysis is completed under the influence of acetyl-CoA acyltransferase (a type of thiolase)^56^. Subsequently, fatty acids that lose two carbon atoms will undergo β-oxidative decomposition. Ultimately, a large amount of acetyl-CoA is produced by complete oxidation. Acetyl-CoA enters the mitochondrial tricarboxylic acid (TCA) cycle for complete oxidative decomposition, to produce carbon dioxide and water, and release large amounts of energy for metabolic activities. The remaining acetyl-CoA from the TCA cycle enters through the liver Lynen cycle, and produces acetoacetate or β-ketobutyrate under the influence of acetyl-CoA acyltransferase, forming further β-hydroxybutyrate via dehydrogenation. This reaction consumes NADH and generates NAD^+^. Acetone and β-hydroxybutyrate are the main components of ketone bodies, and are important presursors in fat synthesis^57^. Under glucose deprivation, the liver can export ketone bodies to muscle tissue for utilisation in skeletal muscle. The current study found that the expression of long-chain acyl-CoA synthetase and acetyl-CoA acyltransferase significantly decreased in the muscle of crisp grass carp. So, we speculated that the fatty acid β-oxidation pathway was downregulated in the muscle of crisp grass carp.

Alpha-oxidation is another fatty acid oxidation pathway, and is the main pathway for the degradation of branched-chain fatty acids^58^. However, its exact mechanism of action is unknown.Previous studies suggested that branched-chain fatty acids form 2-hydroxyacyl-CoA under the influence of long-chain acyl-CoA synthetase. There are two main theoretical speculations for the oxidation pathway after 2-hydroxyacyl-CoA: one is the formation of 2-hydroxy fatty acids via 2-keto fatty acids (α-oxidation mono-oxygenation pathway); and the other is the formation of 2-hydroxy fatty acids under the influence of aldehyde dehydrogenase (α-oxidation dioxygenation pathway)^59^. Subsequently, 2-hydroxy fatty acids are oxidised by *(S)*-2-hydroxy-acid oxidase^60^. In the current study, the expression of long-chain acyl-CoA synthetase, *(S)*-2-hydroxy-acid oxidase and xanthine dehydrogenase or oxidase were decreased in the muscle of crisp grass carp. However, the expression of aldehyde dehydrogenase was increased. Thus, changes in α-oxidation remain unclear.

Our previous study found that the levels of saturated and monounsaturated fatty acids were significantly higher in the muscle of crisp grass carp than in that of ordinary grass carp^61^. This may be related to the decreased fatty acid degradation in the current study. It is speculated that fatty acid degradation and β-oxidation are decreased in the muscle of crisp grass carp with greater muscle hardness. However, the changes in the α-oxidation pathway need to be further investigated.

Compared with ordinary grass carp, the muscle hardness and chewiness were significantly increased, the muscle fibre diameter was decreased and the fibre density was increased in crisp grass carp. Proteomic analysis identified 99 differentially expressed proteins in the muscle of crisp grass carp with increased muscle hardness. Accordingly, a protein–protein interaction network consisting of 56 differentially expressed proteins was generated. Both lipid metabolism and glucose metabolism were closely related in this network. Moreover, these two metabolisms were connected in the muscle fibre composition protein network via the heat shock response, thus forming a protein–protein interaction network of muscle metabolism and muscle structure regulated by plant nutrition. Further analysis of the protein–protein interaction network revealed that muscle fibre hyperplasia was closely related to the protein–protein network of 12 muscle component proteins. Muscle fibre hyperplasia was also accompanied by decreased protein abundance in the fatty acid degradation and calcium signalling pathways. In addition, metabolism via the pentose phosphate pathway decreased after ingestion of faba bean, leading to haemolysis. This finding may be of reference in the prevention and treatment of human g6pd deficiency (“favism”).

In summary, this study investigated the molecular mechanisms of changes in muscle fibres at the protein level in grass carp after being fed with faba bean solely for 100 days. The results of this study provided a new and basic proteomic network for the nutritional regulation of fish muscle. These findings might also offer potential insights for research into the nutritional regulation of food health products on human muscle (e.g., muscle bodybuilding, exercise and disease).

## Materials and Methods

The feeding experiment was carried out at the Dongsheng Aquatic Breeding Base (Zhongshan, Guangdong, China) on fish with an initial mean weight of 1508 ± 72 g (Table 4). A total of 180 fish with this initial weight were randomly divided into ordinary grass carp and crisp grass carp groups, with three replicates per treatment group. They were cultured in six tanks of flow through system (10 × 10 × 2 m), with 30 fish in each tanks. Ordinary grass carp were fed on formulated diet (Tongwei Company, China), and crisp grass carp were fed only on whole faba bean for 100 days. The ingredients of the formulated diet included the following: fish meal, 5 g kg^−1^; soybean meal, 215 g kg^−1^; cottonseed meal, 80 g kg^−1^; rapeseed meal, 200 g kg^−1−1^; wheat flour, 180 g kg^−1^; rice bran, 150 g kg^−1^; lees powder, 50 g kg^−1^; malt root, 50 g kg^−1^; choline chloride, 20 g kg^−1^; mineral mixture 20 g kg^−1^; vitamin mixture, 30 g kg^−1^. The composition of the formulated diet and faba bean were shown in the Table 5.

After 100 days, the final weights were 3445 ± 521 g for crisp grass carp and 3984 ± 360 g for ordinary grass carp (Table 4). Six crisp grass carp and six ordinary grass carp were sampled. Each fish was handled according to the procedures approved by the Malmö-Lund Ethical Committee, and individually euthanised in pH-buffered tricaine methanesulfonate (250 mg L^−1^). The obtained muscle samples were snap-frozen in liquid nitrogen and stored at −80 °C for determination of the muscle hardness and chewiness, and for protein analysis. Muscle samples were fixed in Bouin’s solution for the measurement of the muscle fibre diameter and density.

### Determination of muscle hardness and chewiness, and muscle fibre diameter and density

Muscle hardness and chewiness were determined with a CT3 texture analyser (Brookfield Engineering Laboratories, Inc., Brookfield, USA). We collected a portion of grass carp back muscle (the junction of the fifth dorsal fin and the lateral line scales, 2.0 × 2.0 × 2.0 cm^3^). A P35 cylindrical probe of the CT3 texture analyser was used to test the compression speed at a pre-test speed of 2 mm s^−1^, a post-test speed of 5 mm s^−1^, and a test speed of 1 mm s^−1^. The compression interval was 2 s, with a compression ratio of 25%. Each sample had three replicates, and each replicate was measured three times.

For the determination of the muscle fibre diameter and density, schematic diagrams of muscle paraffin sections were first obtained. The main steps for preparing the muscle paraffin sections were as follows: after sampling (fillet muscle at the junction of the fifth dorsal fin and the lateral line scales was removed and rinsed with saline solution (9 g L^−1^ NaCl)), a 3-mm-thick tissue block was cut from the exposed dorsal white muscle, and a standard histology protocol (Haematoxylin and eosin staining) then carried out^62^. Secondly, the muscle fibre area within a certain field area was measured, and the number of muscle fibres within that field were counted using the DP2-BSW 2.2 software (build 6212, Olympus, Tokyo, Japan). According to the method used^63^, assuming that the muscle fibres are cylindrical, the diameter was calculated according to *s* = π*r*^2^ (where *s* is the muscle fibre area and *r* is the muscle fibre radius). A total of 300 muscle fibres were measured for each sample.

### Blood collection and haematological analyses

Fish were anaesthetised in pH-buffered tricaine methanesulfonate (250 mg L^−1^), and haematological analyses were performed for six fish in each treatment group. Blood samples of 1–1.5 mL were collected from the tail vein, and then 0.5 mL of each blood sample was placed in a vial containing anticoagulant EDTA-K_2_-2H_2_O (2 mg mL^−1^), for complete blood analyses. The white and red blood cell counts, haemoglobin concentration, mean corpuscular haemoglobin concentration, and platelet concentration were measured using a fully automatic haematology analyser (Mek-7222K, Nihon Konden, Tokyo, Japan).

### Preparation of the protein extract

Pooled muscles were homogenised with a Dounce homogeniser (Wheaton Co., Wheaton, IL, USA) in a solution of 6 M guanidine hydrochloride, 500 mM triethylammonium bicarbonate buffer (TEAB, pH 8.3) and 0.1% Triton X-100. Acetone precipitation was then carried out, and the proteins were resuspended in 500 mM TEAB with 1 M urea and 0.1% sodium dodecyl sulfate.

### iTRAQ labelling

The concentrations of muscle proteins were measured with a Quant-iT protein assay kit in a QuBit fluorometer (Invitrogen, Carlsbad, CA, USA). Proteins (100 μg) were used for iTRAQ labelling following the manufacturer’s protocols with an 8-plex iTRAQ kit (Applied Biosystems, Foster, CA, USA). The three control samples were pooled and labelled with 116 tag, and three treated samples were labelled with the 113, 114, and 115 tags. The labelling reaction was quenched by adding 100 μL of water. The four samples were combined and evaporated in a Speedvac Concentrator 5301 (Eppendorf, Hamburg, Germany). Water (100 μL) was then added and evaporated: this step was carried out twice.

### HPLC fractioning

Each labelled protein sample was fractionated on an SCX column using an Ultimate 3000 HPLC system (Dionex, Sunnyvale, CA, USA). The samples were first resuspended in 10 mM KH_2_PO_4_ and 25% acetonitrile (ACN) at pH 2.7 (buffer A), and centrifuged. Next, 250 μL was separated with a 0–100% gradient of buffer B (10 mM KH_2_PO_4_, 0.5 M KCl and 25% ACN). Twenty fractions were collected every minute, and subsequently evaporated to dryness.

### Nano LC-MS/MS

Each fraction was resuspended in 2% ACN and 0.1% HCOOH. About 2.5 μL was separated on a PicoFrit column (BioBasic C18, 75 μm × 10 cm, tip 15 μm; New Objective, Woburn, MA, USA) using an Ultimate 3000 nano-HPLC system (Dionex, Sunnyvale, CA, USA) in tandem with a nano-ESI-QqTOF (QStar Pulsar i, Applied Biosystems, Foster, CA, USA) with an ACN gradient. The information-dependent acquisition (IDA) mode was used with the following parameters: from the 2 s MS spectrum, the two most intense multiply- charged precursor ions (+2 to +4) were selected for 2 s MS/MS spectral acquisitions. The *m/z* values of the precursor ions selected were excluded for 90 s, to avoid their reanalysis. The minimum threshold intensity of the ions was set to ten counts. The ion spray and declustering potentials were 5200 and 50 V, respectively. The collision energies for the gas phase fragmentation of the precursor ions were determined automatically by IDA on the basis of their *m/z* values. The data were acquired and analysed using the Analyst QS version 1.1 software (Applied Biosystems, Foster, CA, USA). Three LC-MS/MS replicates were run. Tandem mass spectra were extracted, and the charge states were deconvoluted and deisotoped by ABI Analyst version 1.1 (Applied Biosystems, Foster, CA, USA). All MS/MS samples were analysed using MASCOT (Matrix Science, London, UK) and X!Tandem version 2007.01.01.1 (The GPM, thegpm.org) with the Scaffold software (Proteome Software, Portland, OR, USA). MASCOT and X!Tandem were set up to search the Ensembl database (release 56) of fish (restricted to *Oryzias latipes, Danio rerio, Takifugu rubripes, Tetraodon nigroviridis*, and *Gasterosteus aculeatus* sequences), assuming the digestion enzyme trypsin. MASCOT and X!Tandem were searched with a fragment ion mass tolerance of 0.50 Da and a parent ion tolerance of 0.50 Da. The methyl methanethiosulfonate of cysteine, *k* + 304 of lysine and *n* + 304 of the N-terminus were specified in MASCOT and X!Tandem as fixed modifications, whereas the oxidation of methionine and the iTRAQ 8-plex of tyrosine were specified as variable modifications. Scaffold was used to probabilistically validate the protein identifications derived from the MS/MS sequencing results using X!Tandem ProteinProphet computer algorithms.

### Quantification

Scaffold Q+ was used to quantify the isobaric tag peptide and protein identifications. Protein identifications were accepted if they could be established with more than 99.0% probability and contained at least two identified peptides. Protein probabilities were assigned by the ProteinProphet algorithm. Proteins that contained similar peptides and could not be differentiated on the basis of MS/MS analysis alone were grouped to satisfy the principles of parsimony. Peptides were quantified using the centroided reporter ion peak intensity. Intrasample channels were normalised on the basis of the median ratio for each channel across all proteins. Multiple isobaric-tagged samples were normalised by comparing the median protein ratios for the reference channel. Protein quantitative values were derived solely from uniquely assigned peptides. The minimum quantitative value for each spectrum was calculated as 0.025% of the highest peak. Protein quantitative ratios were calculated as the median of all peptide ratios of the three runs. Standard deviations were calculated as the interquartile range around the median. Quantitative ratios were log_2_ normalised for final quantitative testing. Differentially expressed proteins were determined using several statistical tests with R software version 2.11.1 (R Foundation for Statistical Computing, Vienna, Austria). The normality of the data was checked with the Shapiro–Wilk test, followed by the *t*-test, the Mann–Whitney test and ANOVA (followed by a Tukey–Kramer *post-hoc* test). *P* < 0.05 was considered statistically significant.

### Data analysis

The original data were processed to filter out repeating proteins. The corresponding protein numbers in the UniProtKB database (http://www.uniprot.org)^64^ were identified according to the protein numbers. When a protein number was not available in the database, the protein was annotated with the closest protein identified by UniProtKB alignment. The MS/MS spectral data were analysed by the Proteinpilot version.3 software (AB Sciex, Foster, CA, USA): (1) protein identification was performed using a confidence threshold of 99% (Proteinpilot unused score of >2.0); (2) the *p* value, representing the probability that the observed ratio is different from 1 by chance, was calculated according to the intensity values of effective peptides in each protein, and using the Student *t*-test method, where *n* represents the number of effective peptides per protein; 3) comparing the experimental and control groups, proteins showing at least twofold differential expression (ratio <0.5 or >2) with *p* < 0.05 were selected and analysed. Gene Ontology (GO) numbers corresponding to each protein were identified by UniProtKB protein annotations (http://www.uniprot.org)^65^. Enrichment analysis of GO functional classification was undertaken on the differentially expressed proteins: a functional class was considered enriched when the number of proteins in the set of differentially expressed proteins accounted for a greater proportion in this GO functional class than the number of proteins in all protein sets, and the chi-square test was *p* < 0.05. The Kyoto Encyclopedia of Genes and Genomes (KEGG) and Kyoto Orthology (KO) protein numbers were obtained via UniProtKB annotations. The annotations of the signalling pathways of differentially expressed proteins in muscle tissues were obtained by searching the KO database (http://www.genome.jp/kegg/ko.html)^66^.

A metabolic pathway was considered enriched when the number of proteins in the set of differentially expressed proteins accounted for a greater proportion in this metabolic pathway than the number of proteins in all protein sets. Protein–protein interaction network analysis was performed as follows. First, blasting alignment of the sequences of differentially expressed proteins and zebrafish sequences (26,163 sequences in total) in the STRING database (V10) (http://string-db.org)^67^,was carried out using the criteria of an *E* value of ≤1 × 10^−20^, a coverage of ≥80% and an identity of ≥80% to obtain zebrafish sequences. Secondly, proteins showing at least twofold differential expression with *p* < 0.05 were identified as differentially expressed proteins. The numbers of zebrafish sequences obtained according to the above criteria were identified. Finally, the data of these two sets of numbers were searched in the STRING database, to restore the interaction map of these proteins. A protein–protein interaction network map was then created using the Medusa software (https://sites.google.com/site/medusa3visualization)^68^.

## Additional Information

**How to cite this article:** Yu, E.-M. *et al*. Proteomic signature of muscle fibre hyperplasia in response to faba bean intake in grass carp. *Sci. Rep.* **7**, 45950; doi: 10.1038/srep45950 (2017).

**Publisher's note:** Springer Nature remains neutral with regard to jurisdictional claims in published maps and institutional affiliations.

## Figures and Tables

**Table 1:** Differentially expressed cytoskeleton, actin cytoskeleton, muscle fibre development, collagen, and calcium-ion-binding proteins.

Protein description	Gene symbol	Accession	Ratio	*p* value
Actin cytoskeleton				
PREDICTED: catenin alpha-1-like	ctnna1	gi|410914176	3.499	0.036
Glycogen synthase 2	gys2	gi|66392235	0.064	0.049
PREDICTED: myosin heavy chain, fast skeletal muscle, partial	mhc	gi|410932313	0.370	0.027
Slow myosin heavy chain 1	smyhc1	gi|87116414	0.236	0.025
PREDICTED: talin-2-like	tln2	gi|410912160	4.742	0.004
Novel slow skeletal troponin T family protein	tnnt	gi|55962571	0.163	0.022
Tropomyosin beta chain	tpm2	gi|50344894	0.194	0.014
Cytoskeleton				
PREDICTED: catenin alpha-1-like	ctnna1	gi|410914176	3.499	0.036
Glycogen synthase 2	gys2	gi|66392235	0.064	0.049
Keratin 18	krt18	gi|380449877	0.236	0.004
PREDICTED: myosin heavy chain, fast skeletal muscle, partial	mhc	gi|410932313	0.370	0.027
Myosin-binding protein C, slow-type	mybpc1	gi|55925504	0.417	0.000
Slow myosin heavy chain 1	smyhc1	gi|87116414	0.236	0.025
PREDICTED: talin-2-like	tln2	gi|410912160	4.742	0.004
Novel slow skeletal troponin T family protein	tnnt	gi|55962571	0.163	0.022
Tropomyosin beta chain	tpm2	gi|50344894	0.194	0.014
Tubulin, alpha 8 like	tuba8l	gi|37362304	2.399	0.033
Novel protein similar to SPEG complex locus (speg)	ENSDARG00000009567	gi|169154737	0.488	0.017
Muscle actin type 1	zgc:86725	gi|291167458	0.215	0.038
Myosin-binding protein C, fast type a	zgc:136545	gi|113951767	2.014	0.015
Muscle fibre development				
Novel protein similar to vertebrate nebulin	neb	gi|94732977	0.353	0.000
Collagen				
Collagen, type I, alpha 1b precursor	col1a1p	gi|41393113	0.457	0.002
Calcium ion binding				
Annexin 11a isoform 2	anxa11	gi|34536830	2.312	0.025
Calreticulin	crt	gi|312603155	0.180	0.013
Myosin, light chain 4	MYL4	gi|41055823	0.217	0.002
Myosin, light chain 10, regulatory	myl10	gi|62955715	0.167	0.005
Phospholipase C-beta 1	plcb1	gi|208342308	2.443	0.038
Troponin C type 1b (slow)	tnnc1b	gi|50344824	0.172	0.001

The proteins sharing similar biological functions were clustered based on similar trends of differential expression. The table lists the protein description, gene symbol, accession number, ratios and *p* values of protein expression. Ratio = (protein reporter ion signal intensity of a protein in the muscle of crisp grass carp)/(protein reporter ion signal intensity of the protein in the muscle of grass carp). The *p* value, representing the probability that the observed ratio is different from 1 by chance, was calculated according to the intensity values of effective peptides in each protein, and using the Student *t*-test method, where *n* represents the number of effective peptides per protein. Ratios were deemed to signify differential expression (*p* < 0.05). These proteins were considered to show a significant upward or downward trend when their expression ratios were <0.5 or >2, respectively.

**Table 2:** Differentially expressed oxidoreductase activity, biosynthetic process, catalytic activity, transferase activity, and ATP-binding proteins.

Protein description	Gene symbol	Accession	Ratio	*p* value
**Oxidoreductase activity**				
Aldo-ketoreductase family 1, member B1	akr1b1	gi|50344750	0.474	0.004
Aldehyde dehydrogenase 9 family, member A1b	aldh9a1b	gi|161784297	87.902	0.019
Aldehyde dehydrogenase 1 family, member L1	aldh1l1	gi|311771613	0.179	0.000
3-Hydroxybutyrate dehydrogenase, type 1	bdh1	gi|147899736	0.050	0.049
RecName: Full = 3-hydroxybutyrate dehydrogenase type 2	bdh2	gi|82193321	0.084	0.029
NADH-cytochrome b5 reductase 2	cyb5r2	gi|113679449	0.391	0.030
Cytochrome P450, family 20, subfamily A, polypeptide 1	cyp20a1	gi|47086981	0.043	0.040
Cytochrome P450 2AD3	cyp2ad3	gi|46243655	0.461	0.043
Uncharacterised protein LOC564675	ENSDARG00000043403	gi|165972501	0.061	0.012
PREDICTED: fatty aldehyde dehydrogenase-like	faldh	gi|410915226	0.316	0.003
PREDICTED: fatty acid synthase-like	fas	gi|410930382	2.228	0.031
Glucose-6-phosphate 1-dehydrogenase	g6pd	gi|213512060	0.027	0.048
Glycerol-3-phosphate dehydrogenase 1b	gpd1b	gi|229366800	9.638	0.042
PREDICTED: glutathione reductase, mitochondrial-like	gsrm	gi|410914397	0.137	0.042
Hydroxyacid oxidase 2	hao2	gi|41053573	0.233	0.049
4-Hydroxyphenylpyruvate dioxygenase a; Key enzyme in the degradation of tyrosine	hpda	gi|82187435	0.205	0.011
Hydroxy-delta-5-steroid dehydrogenase, 3 beta- and steroid delta-isomerase	hsd3b7	gi|44890388	12.589	0.028
NAD(P)-dependent steroid dehydrogenase-like	nsdhl	gi|62955325	0.156	0.014
Phenylalanine hydroxylase	pah	gi|41054599	0.083	0.044
Pyruvate dehydrogenase (lipoamide) alpha 1a	pdha1a	gi|53749653	0.182	0.045
Protein disulfideisomerase associated 4	pdia4	gi|46361720	0.215	0.022
Phytanoyl-CoA dioxygenase domain-containing protein 1	phyhd1	gi|226358555	0.143	0.035
Peroxiredoxin 6	prdx6	gi|55251344	0.112	0.005
PREDICTED: xanthine dehydrogenase/oxidase	xdh	gi|189530915	0.011	0.048
Uncharacterised protein LOC393297	zgc:56585	gi|41056185	0.138	0.031
**Oxidoreductase activity, acting on the aldehyde or oxo group of donors**				
Aldehyde dehydrogenase 9 family, member A1b	aldh9a1b	gi|161784297	87.902	0.019
Aldehyde dehydrogenase 1 family, member L1	aldh1l1	gi|311771613	0.179	0.000
PREDICTED: fatty aldehyde dehydrogenase-like	faldh	gi|410915226	0.316	0.003
Pyruvate dehydrogenase alpha 1a	pdha1a	gi|53749653	0.182	0.045
**Oxidoreductase activity, acting on the CH–OH group of donors, NAD or NADP as acceptor**				
RecName: Full=3-hydroxybutyrate dehydrogenase type 2	bdh2	gi|82193321	0.084	0.029
PREDICTED: fatty acid synthase-like	fas	gi|410930382	2.228	0.031
Glucose-6-phosphate 1-dehydrogenase	g6pd	gi|213512060	0.027	0.048
Glycerol-3-phosphate dehydrogenase 1b	gpd1b	gi|229366800	9.638	0.042
Hydroxy-delta-5-steroid dehydrogenase, 3 beta- and steroid delta-isomerase	hsd3b7	gi|44890388	12.589	0.028
NAD(P) dependent steroid dehydrogenase-like	nsdhl	gi|62955325	0.156	0.014
PREDICTED: xanthine dehydrogenase/oxidase	xdh	gi|189530915	0.011	0.048
**Single-organism biosynthetic process**				
RecName: Full=3-hydroxybutyrate dehydrogenase type 2	bdh2	gi|82193321	0.084	0.029
NADH-cytochrome b5 reductase 2	cyb5r2	gi|113679449	0.391	0.030
RecName: Full=glycine amidinotransferase, mitochondrial	gatm	gi|82187306	0.177	0.012
Glycogen synthase 2	gys2	gi|66392235	0.064	0.049
Hydroxy-delta-5-steroid dehydrogenase, 3 beta- and steroid delta-isomerase	hsd3b7	gi|44890388	12.589	0.028
Isopentenyl-diphosphate delta-isomerase 1	idi1	gi|70887565	0.215	0.038
NAD(P)-dependent steroid dehydrogenase-like	nsdhl	gi|62955325	0.156	0.014
Pyruvate carboxylase	pc	gi|300641377	0.053	0.009
Pyruvate carboxylase, like	pcl	gi|83415094	0.023	0.033
Sigma non-opioid intracellular receptor 1	sigmar1	gi|82209697	0.316	0.022
**Lipid biosynthetic process**				
NADH-cytochrome b5 reductase 2	cyb5r2	gi|113679449	0.391	0.030
Hydroxy-delta-5-steroid dehydrogenase, 3 beta- and steroid delta-isomerase	hsd3b7	gi|44890388	12.589	0.028
Isopentenyl-diphosphate delta-isomerase 1	idi1	gi|70887565	0.215	0.038
NAD(P)-dependent steroid dehydrogenase-like	nsdhl	gi|62955325	0.156	0.014
Sigma non-opioid intracellular receptor 1	sigmar1	gi|82209697	0.316	0.022
**Catalytic activity**				
DIP2 disco-interacting protein 2 homolog B	dip2ba	gi|82185659	0.156	0.041
26 S proteasome non-ATPase regulatory subunit 2	psmd2	gi|41054527	3.767	0.001
Acyl-CoA synthetase long-chain family member 1	zgc:101071	gi|57525836	0.242	0.023
**Transferase activity**				
Acetyl-coenzyme A acyltransferase 1	acaa1	gi|50345072	0.146	0.045
Glutathione *S*-transferase M	gstm	gi|218455223	2.312	0.008
Glutathione *S*-transferase pi 1	gstp1	gi|218455225	3.733	0.023
*N*-Myristoyltransferase 1a	nmt1a	gi|47217841	0.071	0.006
Rho-class glutathione-*S*-transferase	rgst	gi|190410767	0.018	0.025
Cytosolic sulfotransferase 2	sult2st2	gi|148745693	0.108	0.042
**ATP binding**				
Aldo-ketoreductase family 1, member B1	akr1b1	gi|50344750	0.474	0.004
Chaperonin containing TCP1, subunit 5	cct5	gi|49619081	2.421	0.018
Novel protein similar to SPEG complex locus (speg)	ENSDARG00000009567	gi|169154737	0.488	0.017
60 kDa heat shock protein 1	hspd1	gi|315585122	0.126	0.008
PREDICTED: isoleucine–tRNA ligase, mitochondrial-like	ltlm	gi|410925971	4.875	0.031
PREDICTED: myosin heavy chain, fast skeletal muscle, partial	mhc	gi|410932313	0.370	0.027
Pyruvate carboxylase	pc	gi|300641377	0.053	0.009
Pyruvate carboxylase-like	pcl	gi|83415094	0.023	0.033
Proteasome (prosome, macropain) 26 S subunit, ATPase, 3	psmc3	gi|50344782	2.148	0.049
Slow myosin heavy chain 1	smyhc1	gi|87116414	0.236	0.025
Muscle actin type 1	zgc:86725	gi|291167458	0.215	0.038

The proteins sharing similar biological functions were clustered based on similar trends of differential expression. The table lists the protein description, gene symbol, accession number, ratios and *p* values of protein expression. Ratio = (protein reporter ion signal intensity of a protein in the muscle of crisp grass carp)/(protein reporter ion signal intensity of the protein in the muscle of grass carp). The *p* value, representing the probability that the observed ratio is different from1 by chance, was calculated according to the intensity values of effective peptides in each protein, and using the Student *t*-test method, where *n* represents the number of effective peptides per protein. Ratios were deemed to signify differential expression (*p* < 0.05). These proteins were considered to show a significant upward or downward trend when their expression ratios were <0.5 or >2, respectively.

**Table 3:** Differentially expressed membrane, nucleus, ribosome, transporter activity and other GO proteins.

Protein description	Gene symbol	Accession	Ratio	*p* value
**Membrane**				
Complement component C9	ccc9	gi|126045475	2.831	0.038
GTP cyclohydrolase I feedback regulator	gchfr	gi|62286827	8.241	0.042
Stomatin-like protein 2	stom2	gi|223648686	0.104	0.021
Troponin C type 1b (slow)	tnnc1b	gi|50344824	0.172	0.001
**Nucleus**				
DIP2 disco-interacting protein 2 homolog Ba	dip2ba	gi|82185659	0.156	0.041
Unnamed protein product	no	gi|311812832	0.134	0.030
Chaperone protein promoting assembly of the 20 S proteasome as part of a heterodimer with psmg1	psmg2	gi|94732190	0.018	0.047
**Ribosome**				
Ribosomal protein L13	rpl13	gi|44966222	0.081	0.021
40 S ribosomal protein S13	rps13	gi|50344812	0.156	0.010
**Transporter activity**				
Archain 1b	arcn1b	gi|49902633	3.565	0.044
Brain-type fatty-acid-binding protein 7a	fabp7a	gi|8809798	0.325	0.040
PREDICTED: importin-4	ipo4	gi|326679575	0.180	0.038
*N*-Ethylmaleimide-sensitive fusion protein attachment protein alpha	napa	gi|41054285	5.297	0.029
Unnamed protein product	no	gi|47224597	7.178	0.039
Solute carrier family 25 alpha, member 5	slc25a5	gi|41107664	0.081	0.039
**Other GOs**				
Adenosylhomocysteinase	ahcy	gi|40363541	0.093	0.008
Beta-2-microglobulin	b2m	gi|57281691	0.084	0.046
PREDICTED: coiled-coil domain-containing protein 47-like	ccdc	gi|410902989	0.340	0.046
Chymotrypsin B1 precursor	ctrb1	gi|47086795	0.102	0.036
D-Dopachrometautomerase	ddt	gi|50344950	0.077	0.003
REDICTED: elongator complex protein 1	elp5	gi|348511894	6.486	0.034
Family with sequence similarity 82, member B	FAM82B	gi|157311701	0.012	0.038
Fumarylacetoacetase	fase	gi|41054569	0.223	0.042
Fibrinogen gamma polypeptide	fgg	gi|295314912	0.273	0.003
Haemoglobin alpha adult-1	hbaa1	gi|22135554	0.064	0.009
PREDICTED: low-quality protein: heat repeat-containing protein 3-like	hrcp3	gi|410912234	2.168	0.037
Interferon-inducible protein Gig1	ifnip	gi|31580624	12.706	0.036
Karyopherin (importin) beta 3	kpnb3	gi|348536891	2.128	0.039
PREDICTED: hypothetical protein LOC100691952	loc100691952	gi|348539826	0.163	0.047
PREDICTED: uncharacterized protein LOC101068541	loc101068541	gi|410918305	0.219	0.044
PREDICTED: bifunctional protein NCOAT-like	LOC571547	gi|326672078	0.053	0.010
PREDICTED: hypothetical protein LOC794259	loc794259	gi|292619409	6.427	0.034
Metallothionein II	mt2	gi|7579035	0.043	0.039
PREDICTED: pleckstrin homology domain-containing family A member 6-like	PLEKHA6	gi|292618909	0.229	0.040
Peptidyl-prolyl *cis*-*trans* isomerase H	ppih	gi|225707560	2.089	0.024
Protein TFG	tfg	gi|41053365	3.837	0.041
Tetratricopeptide repeat domain 36	ttc36	gi|187471140	0.082	0.008

The proteins sharing similar biological functions were clustered based on similar trends of differential expression. The table lists the protein description, gene symbol, accession number, ratios and *p* values of protein expression. Ratio = (protein reporter ion signal intensity of a protein in the muscle of crisp grass carp)/(protein reporter ion signal intensity of the protein in the muscle of grass carp). The *p* value, representing the probability that the observed ratio is different from 1 by chance, was calculated according to the intensity values of effective peptides in each protein, and using the Student *t*-test method, where *n* represents the number of effective peptides per protein. Ratios were deemed to signify differential expression (*p* < 0.05). These proteins were considered to show a significant upward or downward trend when their expression ratios were <0.5 or >2, respectively.

**Table 4:** Growth performance of grass carp feeding formulated diet and faba bean.

	Control group	Experimental group
Start weights, g (mean ± SD, n=90)	1507 ± 81^a^	1509 ± 62^a^
Final weights, g (mean ± SD, n=90)	3984 ± 360^a*^	3445 ± 521^b*^
Weight gain rate (%)	163.97 ± 14.56^a*^	128.83 ± 37.13^b*^
Specific gain rate (% day^−1^)	0.97 ± 0.06^a*^	0.82 ± 0.16^b*^
Feed conversion ratio	2.27 ± 0.27^b**^	4.32 ± 0.49^a**^

Independent-samples Tukey’s text was carried out for the two independent samples, and *p* and *F* values were accurately calculated (95% confidence levels). Means with different superscripts in the same row are statistically different, *p < 0.05, **p < 0.01, ***p < 0.001.

**Table 5:** The composition of the formulated diet and faba bean.

	Formulated diet	Faba bean
Dry matter (g kg^−1^)	902	870
Crude protein (g kg^−1^)	297	282
Crude lipid (g kg^−1^)	29	12
Ash (g kg^−1^)	120	27
